# Do all roads lead to Oct4? The emerging concepts of induced pluripotency

**DOI:** 10.1016/j.tcb.2013.11.010

**Published:** 2014-05

**Authors:** Aliaksandra Radzisheuskaya, José C.R. Silva

**Affiliations:** 1 Wellcome Trust – Medical Research Council Cambridge Stem Cell Institute, University of Cambridge, Tennis Court Road, Cambridge CB2 1QR, UK; 2 Department of Biochemistry, University of Cambridge, Tennis Court Road, Cambridge CB2 1QR, UK

**Keywords:** reprogramming, cell differentiation, embryonic stem cells, induced pluripotent stem cells, cell state transitions, naïve pluripotency

## Abstract

Pluripotent cells have the potential to differentiate into all of the cell types of an animal. This unique cell state is governed by an interconnected network of transcription factors. Among these, Oct4 plays an essential role both in the development of pluripotent cells in the embryo and in the self-renewal of its *in vitro* counterpart, embryonic stem (ES) cells. Furthermore, Oct4 is one of the four Yamanaka factors and its overexpression alone can generate induced pluripotent stem (iPS) cells. Recent reports underscore Oct4 as an essential regulator of opposing cell state transitions, such as pluripotency establishment and differentiation into embryonic germ lineages. Here we discuss these recent studies and the potential mechanisms underlying these contrasting functions of Oct4.

## Highlights

• Oct4 has unique and diverse functions in reprogramming.
• Oct4 is essential for lineage specification.
• Oct4 regulates multiple contrasting processes of cell identity change.
• Oct4 function may be regulated by cellular context and environment.

## Oct4 in pluripotency

Cell identity is characterised by a stable, unique, self-sustaining gene expression pattern. Cell state transition represents a change from one cell identity to another. Such transitions occur in development during differentiation into progressively specialised cell types, which is accompanied by a gradual restriction of developmental potential. Pluripotency characterises the cells capable of giving rise to all of the cell types of an organism except for extraembryonic tissues 1, 2. In mouse embryos, pluripotent cells emerge in the epiblast during preimplantation development and, shortly after implantation, undergo differentiation into embryonic germ layers and the germline. The pluripotent cells in the preimplantation embryo are considered naïve as opposed to the primed pluripotent cells of the postimplantation embryo, because they have unbiased developmental potential and can give rise to germline-competent chimeras when reintroduced into a blastocyst [3]. The *in vitro* counterparts of this transient naïve pluripotent cell population are ES cells. Similarly to the naïve epiblast, ES cells coexpress the pluripotency markers Esrrb, Nanog, Rex1, Klf4, Klf2, Sox2, Tbx3, Tfcp2l1, and Oct4, have two active X chromosomes in the case of female cells, and can give rise to fully ES cell-derived mice [4].

Although cell state transitions are typically perceived as moving from a less differentiated to a more differentiated state, groundbreaking work by Takahashi and Yamanaka demonstrated that the cell state transition from somatic cells to naïve pluripotency is also possible and can be induced by the overexpression of four transcription factors: Oct4, Sox2, Klf4, and c-Myc [5]. The derived cells were named iPS cells and they have since been obtained from different species and types of somatic cells. Although the method of reprogramming is firmly established, the molecular mechanisms underlying this process remain poorly characterised.

Among the four original reprogramming factors, the POU family transcription factor Oct4 appears to be the most important pluripotency regulator. Oct4 was found to be required for the formation of the naïve epiblast, because the inner cell mass of Oct4-null embryos lacks pluripotent characteristics [6]. In addition, abrogation of Oct4 expression in ES cells leads to their differentiation along the trophoblast lineage [7]. Although the SRY-related HMG-box transcription factor Sox2 has also been shown to possess a loss-of-function phenotype similar to that of Oct4 in both embryos [8] and ES cells [9], ectopic expression of wild type levels of Oct4 can rescue the Sox2-null phenotype in ES cells [9]. This result indicates that Oct4 activation may be the sole essential function of Sox2 in ES cell self-renewal. In contrast to Oct4 and Sox2, other pluripotency factors seem to be individually dispensable for the maintenance of the naïve pluripotent state 10, 11, 12, 13, 14, 15. Overexpression of Nanog [16], Esrrb [17], Klf4, Klf2 [18], Tfcp2l1 [15], and Tbx3 [19] leads to enhanced self-renewal of ES cells, illustrating a positive effect on the pluripotent network. In turn, overexpression of Oct4 or Sox2 leads to spontaneous ES cell differentiation 7, 20.

Recent studies are revealing novel aspects of the biological functions of Oct4. Particularly, Oct4 was found to regulate seemingly opposite processes of cell identity change: the induction of pluripotency from somatic cells, pluripotent cell differentiation into embryonic lineages, and transdifferentiation – that is, the conversion of one somatic cell type into another without a common progenitor. In this review, we discuss these recent studies and the potential molecular mechanisms underlying these contrasting roles of Oct4 and propose that Oct4 is an essential regulator of cell state transitions in development.

## Oct4 in reprogramming

In addition to being an essential regulator of pluripotency, Oct4 is also central to nuclear reprogramming. Oct4 overexpression is sufficient to induce pluripotency when using somatic cell types expressing canonical reprogramming factors endogenously 21, 22, 23, 24 or when in combination with small molecules 25, 26, 27. However, reprogramming with Oct4 alone exhibits decreased efficiency and delayed kinetics. Significantly, exogenous Oct4 was shown to be replaceable in initiating reprogramming; however, most of the factors and small molecules demonstrated to do this (Table 1) act by reactivating the endogenous *Oct4* locus. The nuclear receptors Nr5a1 and Nr5a2, which were shown to induce pluripotency in the absence of Oct4 [28], bind regulatory regions of the *Oct4* gene and activate its expression in ES cells and embryonic carcinoma (EC) cells 29, 30. Tet1 was recently demonstrated to replace exogenous Oct4 in reprogramming by promoting 5mc–5hmc conversion at the *Oct4* regulatory regions and, thereby, to contribute towards the reactivation of the endogenous locus [31]. In addition, Tet1 together with Nanog was found to synergistically activate the endogenous *Oct4* locus in reprogramming intermediates [32]. Oct4 is also not required to initiate reprogramming when the following transgene combinations are used: (i) Sox2, Sall4, Nanog, Klf4, C-Myc; (ii) Lin28, Sall4, Esrrb, Nanog, Klf4, c-Myc; (iii) Lin28, Sall4, Esrrb, Nanog; (iv) Lin28, Sall4, Esrrb, Dppa2; (v) Lin28, Sall4, Ezh2, Nanog, Klf4, c-Myc [33]; and (vi) Sall1, Sall4, Utf1, c-Myc, Nanog [34]. Notably, all of the combinations contain Sall4 as a reprogramming factor and Bayesian network analysis positions Sall4 upstream of Oct4 in the sequence of events leading to the establishment of naïve pluripotency [33]. Moreover, Sall4 was previously reported to positively affect the expression of Oct4 in both mouse and human ES cells 35, 36, suggesting that Sall4 may activate *Oct4* during reprogramming. Importantly, it was recently demonstrated that an overexpression of a custom-made transcription activator targeting the *Oct4* enhancer leads to strong activation of the endogenous *Oct4* locus and efficient iPS cell generation in the absence of Oct4 in the reprogramming cocktail [37]. This finding proves that any factor capable of activating Oct4 expression could replace it in reprogramming.

**Table 1:** Reprogramming cocktails without Oct4

Alternative reprogramming cocktail	Somatic cell type	Refs	Evidence of acting through endogenous Oct4?
KSM + BIX-01294	Primary mouse fetal neural progenitor cells	[64]	Yes
KSM + Nr5a1 KSM + Nr5a2	Mouse embryonic fibroblasts	[28]	Yes
KSM + Tet1	Mouse embryonic fibroblasts	[31]	Yes
KSM + Sall4 + Nanog KM + Lin28 + Sall4 + Esrrb + Nanog KM + Lin28 + Sall4 + Ezh2 + Nanog Lin28 + Sall4 + Esrrb + Nanog Lin28 + Sall4 + Esrrb + Dppa2	Mouse embryonic fibroblasts	[33]	Yes
KSM + Gata3	Mouse embryonic fibroblasts Mouse adult dermal fibroblasts Mouse gastric epithelial cells Mouse keratinocytes	[68]	No
KSM + Sox7 KSM + Pax1 KSM + Gata4 KSM + CEBPa KSM + HNF4a KSM + GRB2 KM + Gata3 + Sox1 KM + Gata3 + Sox3 KM + Gata6 + Sox1 KM + Gata6 + Sox3 KM + Pax1 + Sox1 KM + Pax1 + Sox3	Mouse adult dermal fibroblasts		
KSM + Gata6	Mouse adult dermal fibroblasts Mouse keratinocytes		
KM + Gata6 + Geminin	Mouse adult dermal fibroblasts Mouse embryonic fibroblasts		
KS^VP^M + Gata3^VP^	Primary human foreskin fibroblasts	[69]	Yes
KSM + E-cadherin	Mouse embryonic fibroblasts	[54]	No
KSM + forskolin KSM + 2-methyl-5-hydroxytryptamine KSM + D4476	Mouse embryonic fibroblasts	[118]	No
VPA + CHIR99021 + 616452 + tranylcypromine + forskolin + DZNep	Mouse embryonic fibroblasts Mouse neonatal fibroblasts Mouse adult fibroblasts Adipose-derived stem cells		
KSM + human Oct4 KSM + *Xenopus* Oct91 KSM + medaka Pou2 KSM + axolotl Oct4 KSM + axolotl Pou2	Mouse embryonic fibroblasts	[43]	Act by direct substitution of Oct4 due to structural and functional homology
KSM + axolotl Oct4 KSM + axolotl Pou2 KM + axolotl Oct4 + axolotl Sox2 KM + axolotl Pou2 + axolotl Sox2	Primary human skin fibroblasts		
KSM + A-OD3 (TALE-based designer transcriptional activator of Oct4)	Mouse embryonic fibroblasts	[37]	Yes

The table lists all studies that have reported reprogramming cocktails not containing Oct4. Abbreviations: K, Klf4; S, Sox2; M, c-Myc; ^VP^, indicates a protein fused to a transcriptional activator VP.

Whereas Sox2, Klf4, and c-Myc can be replaced by their family members during reprogramming, octamer-binding POU family members Oct1 and Oct6 cannot replace Oct4 [38]. In addition, Oct1, Oct2, and Oct6 cannot sustain pluripotency in Oct4-null mouse ES cells [39]. The unique requirement for Oct4 function in reprogramming, as opposed to other POU family members, was recently attributed to the linker region connecting the two DNA-binding domains of the protein [40]. Mutation of the key amino acids in this linker led to complete abrogation of Oct4 reprogramming ability, while having no effect on its DNA binding, transactivation potential, or nuclear localization. Further analysis demonstrated that these key amino acids are located at the surface of the protein and are potentially involved in the recruitment of epigenetic modifiers to Oct4 target genes [40].

Several reports investigated the evolutionary conservation of Oct4 as a reprogramming factor. According to the current view, *Oct4* (also known as *Pou5f1*) and its paralog *Pou5f3* (also known as *Pou2*) arose by gene duplication at least as early as the last common ancestor of gnathostomes [41]. *Oct4* was subsequently lost in teleost fish, anurans, crocodilians, and birds. In turn, *Pou5f3* was lost in squamate reptiles and eutherian mammals, whereas marsupials, monotremes, urodeles, coelacanths, and turtles retained both genes 41, 42. Tested *Oct4* orthologues, human and axolotl, were shown to replace exogenous mouse Oct4 in reprogramming [43]. Among the *Pou5f3* orthologues, the ability to initiate reprogramming in the mouse system was found for medaka, axolotl, and *Xenopus* but not zebrafish [43]. Importantly, human, axolotl and *Xenopus* but not zebrafish Oct4 homologues can also maintain ES cell self-renewal in the absence of endogenous Oct4 44, 45. In turn, overexpression of mouse or human Oct4 in combination with other Yamanaka factors in avian, zebrafish, and fly somatic cells leads to the upregulation of endogenous pluripotency gene homologues and the formation of partially reprogrammed cells 46, 47. Together, these results demonstrate functional conservation between Oct4 homologues.

In summary, Oct4 is a powerful reprogramming factor with evolutionarily conserved functions that are non-redundant within the POU gene family.

## Molecular mechanisms of Oct4 in reprogramming

Despite its established importance for reprogramming, the precise mechanism of Oct4 action during this process remains unclear. Experimental evidence suggests that Oct4 participates in the induction of the mesenchymal-to-epithelial transition (MET) and in the derepression of somatic cell chromatin. In addition, Oct4 in cooperation with Sox2 was proposed to prevent the acquisition of alternative cell states during reprogramming. Furthermore, Oct4 dose and cellular localisation were proposed as important parameters of successful reprogramming.

### Facilitating the MET

**Figure 1:**
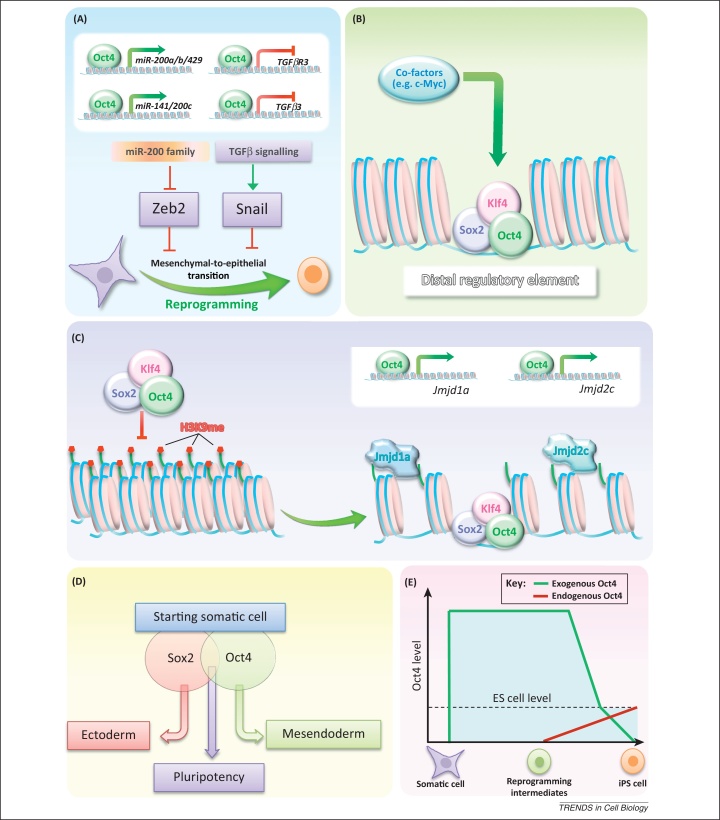
Potential Oct4 mechanisms in reprogramming. **(A)** Oct4 facilitates the mesenchymal-to-epithelial transition (MET) via repression of *TGFβR3* and *TGFβ3* and activation of the *miR-200* family of miRNAs, which lead to repression of the epithelial-to-mesenchymal transition (EMT) regulators Snail and Zeb2, respectively. **(B)** During reprogramming, Oct4, Sox2, and Klf4 act as ‘pioneer’ factors. They bind closed chromatin at distal gene regulatory regions in somatic cells before gene activation and recruit other transcription factors and chromatin modifiers that can then facilitate gene reactivation. **(C)** Histone H3 lysine 9 (H3K9) methylation at the regulatory regions of pluripotency genes presents a major roadblock to reprogramming, because it prevents the binding of reprogramming factors. Oct4 may be involved in overcoming this barrier via activation of the H3K9 demethylases Jmjd1a and Jmjd2c. **(D)** The counterbalance of the lineage-specifying forces of Oct4 and Sox2 precludes the acquisition of alternative cell states in reprogramming favouring the establishment of pluripotency. **(E)** Oct4 is required at high levels throughout the reprogramming process. However, an embryonic stem (ES) cell level of Oct4 expression needs to be achieved at the latest reprogramming stages for a naïve pluripotent state to be established.

Acquisition of an epithelial phenotype by mesenchymal cells, or the MET, is a hallmark of reprogramming initiation [48]. The importance of this process is illustrated by the abrogation of reprogramming in response to MET inhibition and by its enhancement on MET induction 49, 50, 51, 52. All four Yamanaka factors are involved in different aspects of MET regulation. Oct4 was shown to downregulate the epithelial-to-mesenchymal transition (EMT) regulator Snail via the repression of *Tgfβ3* and *TgfβR3* [52] and, together with Sox2, to activate a specific cluster of the miR-200 miRNA family that in turn represses the expression of the EMT regulator Zeb2 [53], thereby facilitating the MET (Figure 1A). Interestingly, overexpression of the MET regulator E-cadherin was shown to replace exogenous Oct4 in reprogramming [54]. This substitution, however, cannot be completely explained by compensation for the role of Oct4 in MET induction, because chemical inhibition of the EMT cannot replace Oct4 in the reprogramming cocktail 49, 50. E-cadherin overexpression may also favour reprogramming via β-catenin sequestration at the membrane and, thereby, inhibition of canonical Wnt-signalling, because Wnt signalling inhibition was shown to facilitate the initial reprogramming stages [55]. However, it is unknown whether this mechanism is connected to endogenous *Oct4* activation during reprogramming. In addition, E-cadherin was recently identified as an important downstream Oct4 effector in establishing cell adhesion properties required to maintain a pluripotent state [56], which may represent another Oct4 mechanism in reprogramming.

### Overcoming epigenetic barriers

According to the current view, reprogramming initiation represents a stochastic process during which reprogramming factors bind the genome promiscuously and act as ‘pioneer’ factors opening up the repressed chromatin and recruiting other transcriptional activators and chromatin remodellers as well as the transcriptional machinery 57, 58, 59 (Figure 1B). Indeed, most of the genomic sites bound by Oct4, Klf4, and Sox2 at the initiation of reprogramming correspond to distal regulatory elements of genes embedded in repressed chromatin in fibroblasts [58]. Some derepression events may eventually occur leading to the activation of early pluripotency genes and, therefore, progression towards the pluripotent state 57, 60. In support of Oct4 pioneer activity, its overexpression in differentiated cells was shown to be sufficient to generate nucleosome-depleted regions at the unmethylated *Nanog* and *Oct4* regulatory sequences leading to basal gene reactivation [61].

Despite the pioneer activity of reprogramming factors, the chromatin of most pluripotency-associated genes is initially inaccessible for binding, which was recently attributed to the presence of the histone H3 lysine 9 trimethylation (H3K9me3) mark [58]. Consistently, global H3K9me3 depletion enhances reprogramming 58, 62. Because Oct4 was demonstrated to be a potent activator of the H3K9 demethylase *Jmjd1a* and *Jmjd2c* genes in mouse ES cells [63], it could also be involved in surpassing this epigenetic roadblock in reprogramming (Figure 1C). Jmjd1a and Jmjd2c are known to maintain Tcl1 and Nanog expression in ES cells through H3K9 demethylation at their regulatory regions [63]. Moreover, the expression of these two H3K9 demethylases increases during reprogramming [5] and their knock down inhibits efficient iPS cell generation [62]. Also in support of this idea, exogenous Oct4 can be replaced in the reprogramming cocktail by the H3K9 methylase G9a inhibitor BIX-01294 [64]. This may also occur at least in part through reactivation of endogenous *Oct4*, because G9a is thought to participate in heterochromatinisation of the *Oct4* locus [65]. However, in the absence of G9a, partial DNA methylation of the *Oct4* locus and *Oct4* repression still occur during ES cell differentiation [66], suggesting that additional mechanisms contribute towards Oct4 repression.

### Seesaw model of reprogramming

Recently, a ‘seesaw reprogramming and pluripotency model’ was proposed suggesting that pluripotency could be established and maintained by counterbalancing the mutually exclusive lineage-specifying forces of Oct4 and Sox2 67, 68, 69 (Figure 1D). Particularly, Oct4 could inhibit the ectodermal programme induced by Sox2. To counterbalance, Sox2 could inhibit the mesendodermal programme induced by Oct4. This model was proposed based on several observations. First, it was recently demonstrated that some mesendoderm-specific transcription factors (e.g., Gata6, Gata4, Sox7, Pax1, CEBPa, HNF4a) can replace exogenous Oct4 in the initiation of reprogramming, whereas some ectoderm-specific transcription factors (e.g., Sox1, Sox3, Otx2, Pax6, Gmnn) can replace exogenous Sox2 68, 69. Second, Oct4 overexpression in ES cells in serum/leukaemia inhibitory factor (LIF) culture conditions was found to induce the predominant formation of mesendodermal lineages [7]. Third, Sox2 promotes neuroectodermal differentiation of pluripotent cells 70, 71 and drives bipotential axial progenitors to a neuroectodermal cell fate in the mouse embryo [72].

The seesaw model implies the existence of tight control of the Oct4/Sox2 ratio, with deviations from such balance directing cells into alternative fates in the context of both reprogramming and pluripotent cell maintenance 67, 68, 69. Indeed, the importance of Oct4 and Sox2 levels for reprogramming and pluripotency maintenance is well established 7, 9, 20, 73, 74, 75, 76, 77, 78, 79, 80. However, alterations of these levels in ES cells do not confirm the proposed idea of a balance shift towards a particular lineage-specifying force. Thus, pluripotent cells can be maintained with low levels of Oct4 and wild type levels of Sox2 without upregulating neuroectoderm markers and instead demonstrating enhanced self-renewal 80, 81. In addition, Sox2 loss in ES cells can be rescued by ectopic expression of wild type levels of Oct4 [9]. Also, Sox2 overexpression in ES cells can generate a mixture of lineages including trophectoderm, mesoderm, and neuroectoderm [20]. Moreover, the original study showing that Oct4 overexpression induces predominant mesoderm and endoderm differentiation in ES cells [7] was performed in the presence of serum containing BMP4, a powerful inhibitor of neuroectoderm differentiation 82, 83. Using the same Oct4 overexpression system but under neural differentiation conditions, Oct4 accelerated neural fate acquisition [84]. Furthermore, overexpression of Oct4 fused to a strong transactivation domain does not induce the expression of mesendodermal genes and does not cause differentiation of ES cells [85]. An alternative explanation for the capacity of lineage-specific master transcriptional regulators to replace conventional reprogramming factors could be their ability to act as pioneer factors. Indeed, it was demonstrated that Gata and HNF factors possess a strong ability to open repressive chromatin 59, 86. This may suggest that Oct4 and other reprogramming factors can be replaced as initiators of reprogramming by any transcription regulator or chromatin remodeller with pioneer activity. Another explanation for pluripotency induction with lineage specifiers could be their ability to upregulate endogenous pluripotency genes. In support of this idea, Gata3 was shown to activate endogenous *Oct4* in reprogramming [69], whereas Gmnn, an ectoderm specifier used to replace Sox2 in reprogramming [68], was reported to be an important positive regulator of pluripotency genes in ES cells [87]. To further evaluate the seesaw reprogramming model, one should compare the genomic occupancy of Oct4 and Sox2 with that of lineage specifiers during reprogramming and analyse the effect of lineage specifiers on the upregulation of the pluripotency network.

In summary, although there may be a place for a seesaw mechanism in pluripotent state regulation, it is unlikely to be a major determinant of cell fate.

### Oct4 levels and cellular localisation in reprogramming

The functions of Oct4 in reprogramming are dose dependent (Figure 1E). Several lines of evidence indicate that transgenic Oct4 must be highly expressed in somatic cells and reprogramming intermediates for successful reprogramming 76, 77, 78, 79, 80, 88, 89. Low levels of transgenic Oct4 led to the generation of iPS cells with aberrant methylation of the Dlk-Dio3 locus, tumourigenicity in chimeric mice, and low capacity of tetraploid complementation. Importantly these could be corrected by increasing the levels of exogenous Oct4 in reprogramming [76]. Another study reported that reprogramming intermediates with low levels of Oct4 are refractory to pluripotency induction, but can be rescued by increasing the OSKM transgene copy number [89]. Therefore, the presence of high Oct4 levels at the initial reprogramming stage may assist in opening chromatin, increasing the chances of reactivating early pluripotency genes.

Recently, it was shown that an ES cell level of Oct4 must be achieved at the late stages of reprogramming for cells to enter the pluripotent cell state [80]. This requirement specifically corresponds to the establishment of pluripotency, because reduced Oct4 levels are compatible with pluripotent cell self-renewal [80]. Notably, endogenous Oct4 activation is not a predictor of pluripotency acquisition, because Oct4 is activated earlier than most of the core pluripotency genes during reprogramming 33, 73, 88, 90. However, the reported reactivation of endogenous Oct4 may have been only partial and thus insufficient for reprogramming completion.

Besides expression levels, the cellular localisation of Oct4 may also have biological importance. It was shown that an Oct4 mutant actively exported from the nucleus can rescue self-renewal of Oct4-null ES cells, but inefficiently induces reprogramming [91]. Interestingly, the same study also described the weaker reprogramming capacity of an Oct4 mutant fused to a strong nuclear localisation signal. Although this result could be explained by the reduced transactivation ability of this mutant [91], the idea that Oct4 may have functions in the cytoplasm is intriguing. Oct4 was shown to be a nucleocytoplasmic shuttling protein 91, 92 and was found in a complex with β-catenin at the membrane of ES cells [93]. However, the precise function of this complex and whether this occurs during reprogramming is unknown.

## Oct4 in cell differentiation

In addition to its role in pluripotency establishment, Oct4 was recently implicated in a contrasting cell state transition; that is, cell differentiation. It was proposed to be involved in the acquisition of extraembryonic endoderm cell fate and in pluripotent cell differentiation into embryonic lineages.

Oct4 was shown to orchestrate the patterning of primitive endoderm during early mouse development by simultaneous activation of multiple primitive endoderm genes [94]. In this study, Oct4-null embryos lost the capacity of Gata6 expression by E4.0 and failed to acquire expression of Sox17, Sox7, and platelet-derived growth factor receptor α (Pdgfrα), defining markers of the primitive endoderm lineage [94]. Another recent study described the cooperation of Oct4 with Sox17 for the activation of endoderm-specific genes during *in vitro* specification of extraembryonic endoderm [95].

Oct4 overexpression studies in mouse ES cells suggest that, depending on the culture environment used, increases in Oct4 levels could induce or enhance cell differentiation into various cellular lineages 7, 70, 80, 84, 96. By contrast, Oct4 overexpression in human ES cells does not lead to spontaneous differentiation or loss of pluripotent cell identity [71]. Instead, Oct4-overexpressing human ES cells exhibit enhanced endoderm and decreased neural differentiation capacity when placed in corresponding differentiation conditions [71]. It should be noted, however, that overexpression-induced differentiation does not indicate a requirement for Oct4 for specification of the formed lineages or proves a negative role of Oct4 in the specification of certain lineages, because overexpression phenotypes could represent neomorphic effects. In fact, when Oct4 is expressed constitutively at a wild type ES cell level in pluripotent cells, they can efficiently enter embryonic development and differentiate into progenitors of all three embryonic lineages and germline in mouse embryos without obvious bias [80]. This is somewhat opposed to both the hypothesis that Oct4 may act as a blocker of certain cellular lineages 70, 71, 97 and the seesaw model for pluripotency and reprogramming 67, 68, 69. Importantly, it was recently demonstrated that mouse iPS cells and ES cells with decreased Oct4 levels can efficiently sustain self-renewal but are incapable of germ layer differentiation both *in vitro* and *in vivo* 80, 81. This proves the requirement of Oct4 for pluripotent cell differentiation. Importantly, the pattern of Oct4 expression in the embryo agrees with these newly uncovered roles in cell differentiation. In addition to pluripotent and germ cells, Oct4 is expressed in the primitive endoderm of the blastocyst stage 98, 99 and in the progeny of all germ layers until the late somite stage 100, 101. Moreover, using conditional Oct4 knockout it was recently demonstrated that Oct4 is required for postimplantation mouse development [102].

How can Oct4 govern such different processes as pluripotency establishment, maintenance, and cell differentiation? Most probably the answer to this seeming paradox lies in the instructive role of the culture environment and cellular context. Oct4 together with Nanog and Sox2 comprises the core of the pluripotency network [103]. These master transcription factors together with Klf4, Esrrb, and Mediator were found to co-occupy the recently classified super-enhancers, which are regulatory sequences found associated with genes involved in the maintenance of ES cell identity [104]. Counterintuitively, under differentiation-inducing culture conditions, Oct4 participates in the repression of pluripotency genes [80]. It is known that the regulatory regions of pluripotency genes are also bound by transcriptional repressor complexes (e.g., Lsd1–NURD) [105]. Also, different Oct4 interactome studies in ES cells demonstrated its interaction with chromatin remodelling complexes, including Lsd1 and NURD 106, 107, 108. Thus, on stimulation with a differentiation inducer, Oct4 may repress pluripotency genes via its association with chromatin repressor complexes. This is consistent with Oct4 typically being downregulated later than other naïve pluripotency-associated genes during ES cell differentiation 80, 109, 110. In support of this view, it was demonstrated that Lsd1 and NURD are not required for the maintenance of ES cell self-renewal, but are important for ES cell differentiation 111, 112, 113. Thus, it will be of future interest to assess the genomic occupancy of both Oct4 and chromatin repressor complexes at early stages of ES cell differentiation.

In addition to its potential role in the silencing of pluripotency genes on induction of cell differentiation, Oct4 is possibly involved in the upregulation of certain lineage-specific genes, as was observed for extraembryonic endoderm specification 94, 95. However, the continuous requirement for Oct4 expression and the extent of Oct4 involvement in embryonic lineage differentiation requires further investigation. In particular, it would be important to address whether Oct4 has specific targets depending on the cell lineage being acquired. This could indicate whether Oct4 may act as a pioneer factor during cell differentiation, providing competence for the establishment of new gene expression patterns in development. This would unite the seemingly contradictory roles of Oct4 in the acquisition and loss of pluripotency.

Taken together, these results describe Oct4 as an essential pan-regulator of cell commitment, potentially involved in silencing of the pluripotency programme and the establishment of lineage-specific gene expression identities instructed by environmental signals.

## Oct4 in transdifferentiation

In addition to its described roles in reprogramming, pluripotent cell self-renewal, and differentiation, Oct4 may also induce transdifferentiation. It has been proposed that human fibroblasts can transdifferentiate into multipotent haematopoietic progenitors when Oct4 is ectopically expressed in fibroblasts treated with cytokines [114]. Furthermore, overexpression of Oct4, c-Myc, Sox2, and Klf4 in combination with a culture environment supportive of the target cell lineage allowed the conversion of mouse fibroblasts into contracting patches of cardiomyocytes [115], neural progenitors [116], and neural stem cells [117]. In all of these studies, the authors provide experimental evidence to exclude the possibility of Oct4 and other Yamanaka factors transiently inducing pluripotent cells that could later differentiate. However, a more extensive investigation utilising cells unable to establish pluripotency or using permanent labelling of cells that have passed through a pluripotent state is required to prove transdifferentiation. The possibility that Oct4 and other Yamanaka factors may induce transdifferentiation of various somatic cell types is a further indication of their pioneer activity in initiating the process that subsequently leads to a cell state change.

## Concluding remarks

**Figure 2:**
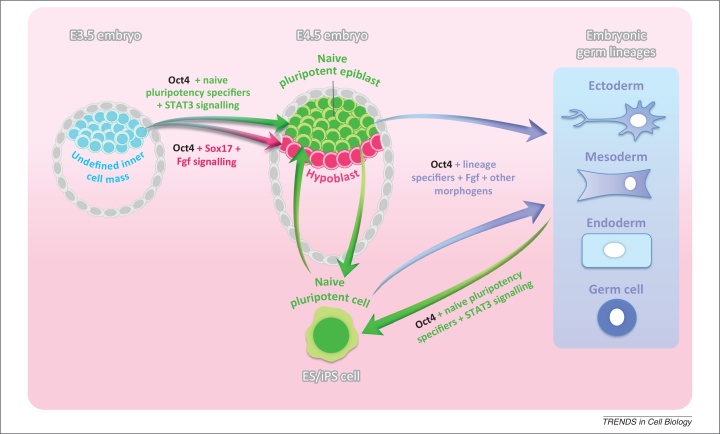
Oct4-controlled cell state transitions. Oct4, together with naïve pluripotency specifiers (Nanog, Klf4, Esrrb, Sox2) and STAT3 signalling, governs the formation of the naïve pluripotent cell compartment in mouse E4.5 blastocysts and directs the reprogramming of somatic cells into a naïve pluripotent cell state. Together with Sox17 and Fgf signalling, Oct4 regulates specification of the primitive endoderm layer (hypoblast) at the blastocyst stage. Under differentiation-inducing culture conditions (on stimulation with Fgf and other morphogens), Oct4 in combination with lineage specifiers drives pluripotent cell commitment into all embryonic germ lineages.

Oct4 is an essential transcriptional regulator with multiple and diverse functions during different stages of reprogramming, pluripotency maintenance, cell differentiation, and transdifferentiation (Figure 2). Although numerous recent studies demonstrate the replacement of exogenous Oct4 by other factors and compounds during reprogramming, most Oct4 substitutions appear to act by upregulating the endogenous *Oct4* locus (Table 1). This underscores the importance of Oct4 in reprogramming, and future investigations should focus on identifying and investigating its downstream functions.

Only recently was Oct4 implicated in cell differentiation, calling for a revaluation of our views on how the pluripotent state is controlled and how commitment is activated. To date, the important differentiation-inducing signals have been identified and we have some knowledge of what chromatin regulators participate in the establishment of new gene expression patterns during differentiation. However, the mechanisms of interconnection between these ‘inducers’ and ‘executioners’ of cell differentiation remain unknown. Could Oct4 be involved in responding to differentiation-inducing stimuli by bringing about specificity to the activity of epigenetic regulators? This idea is intriguing and of interest to investigate in the future.

In conclusion, we have summarised our current understanding of the roles of Oct4 in various aspects of reprogramming, pluripotency, and cell differentiation. The emerging concept is that Oct4 is not merely a master pluripotency self-renewal factor, but, in addition, a key facilitator of cell state transitions occurring during cell differentiation, reprogramming, and transdifferentiation (Figure 2). In the future it would be important to define Oct4 targets and interaction partners during different cell state transitions. This will help in understanding how Oct4 function is regulated by the cellular context and/or extracellular signalling and what role Oct4 plays at different stages of reprogramming and in the initial specification of different lineages.
